# Ivermectin inhibits canine mammary tumor growth by regulating cell cycle progression and WNT signaling

**DOI:** 10.1186/s12917-019-2026-2

**Published:** 2019-08-02

**Authors:** Hongxiu Diao, Nan Cheng, Ying Zhao, Huihao Xu, Haodi Dong, Douglas H. Thamm, Di Zhang, Degui Lin

**Affiliations:** 1 Department of Veterinary Clinical Science, College of Veterinary Medicine, China Agriculture University, Beijing, China; 2 Department of Clinical Sciences, College of Veterinary Medicine and Biomedical Sciences, Colorado State University, Fort Collins, CO USA

**Keywords:** Dog, Cancer, β-Catenin

## Abstract

**Background:**

Mammary gland tumor is the most common spontaneous tumor in intact female dogs, and its poor prognosis remains a clinical challenge. Ivermectin, a well-known anti-parasitic agent, has been implicated as a potential anticancer agent in various types of human cancer. However, there are no reports evaluating the antitumor effects of ivermectin in canine mammary tumor. Here, we investigated whether ivermectin was able to inhibit canine mammary tumor development and explored the related mechanisms.

**Results:**

Ivermectin inhibited the growth of canine mammary tumor cell lines in a dose- and time-dependent manner. The antitumor effects induced by ivermectin were associated with cell cycle arrest at G1 phase via down-regulation of CDK4 and cyclin D1 expression, with no significant induction of apoptosis. Furthermore, significantly reduced β-catenin nuclear translocation was observed after treatment with ivermectin, resulting in the inactivation of WNT signaling. Consistent with the results in vitro, a significant suppression of tumor growth by ivermectin was observed in canine mammary tumor xenografts.

**Conclusion:**

Ivermectin, as a promising anti-cancer agent, inhibits the growth of canine mammary tumor by regulating cell cycle progression and WNT signaling.

**Electronic supplementary material:**

The online version of this article (10.1186/s12917-019-2026-2) contains supplementary material, which is available to authorized users.

## Background

As in women, mammary gland tumor (MGT) is the most common tumor in intact female dogs [1], and a higher incidence of malignant MGT in spayed female dogs deserves more attention [2]. Around 20–80% of canine mammary tumors are diagnosed as malignant [2]. At present, multiple approaches (surgical resection, chemotherapy or their combinations) are utilized, but recurrence and/or metastases remain problematic in a subset of patients [3]. Thus, there is a need for novel potential therapeutic agents to inhibit the growth of MGT and prolong the patient’s life.

Drug repurposing has become an attractive approach due to the known pharmacological and pharmacokinetic properties of approved drugs [4, 5]. Ivermectin is a well-known anti-parasitic agent used to treat a variety of canine parasitic infestations. The mechanism of the action of ivermectin in parasites is due to blockade of parasite chloride channels [6]. Currently, ivermectin has been implicated as a potential anticancer agent in different tumour types (e.g. colon cancer, breast cancer and glioblastoma); putative mechanisms of action have been variable, and include inhibition of WNT-TCF pathway activity, blocking the PAK1/Akt axis, and inducing mitochondrial dysfunction [7–9]. Further studies are needed to explore the detailed molecular mechanisms of ivermectin-associated anti-tumor activity.

A hallmark of cancer is accelerated rates of cell proliferation, which is tightly intertwined with cell cycle and apoptosis regulation [10, 11]. Cell proliferation is a natural process regulated by checkpoints, but these regulators are often altered in cancer cells [12]. These alterations allow cancer cells to acquire the abilities to evade the control of cell cycle and obtain unlimited replication potential [13]. Therefore, targeting cell cycle checkpoints has become popular in human cancers [14].

Based on the aforementioned data, we sought to evaluate ivermectin as a potential anti-tumor drug in canine mammary tumor cells in vitro and in a xenograft model. We found that ivermectin inhibited canine mammary tumor growth by regulating cell cycle progression and the WNT/β-catenin signalling pathway.

## Results

### Ivermectin inhibits cell proliferation

**Fig. 1:**
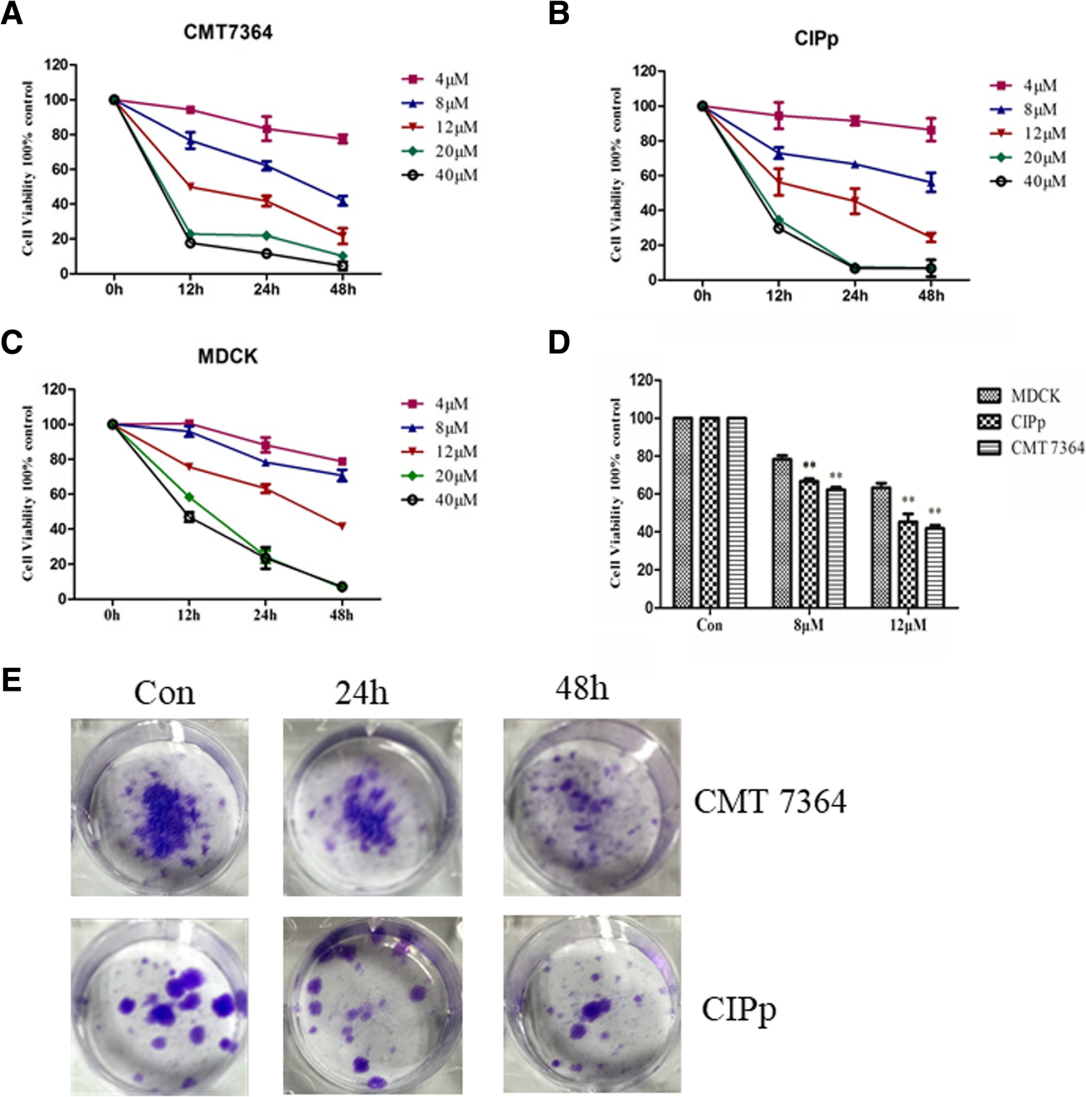
Ivermectin inhibits the growth of canine mammary cancer cells. Cell viability was analyzed using CCK-8 in (**a**) CMT7364, **b** CIPp cells, and (**c**) MDCK cells. **d** Cell viability was detected following a 24-h exposure to 8 μM and 12 μM ivermectin. Data represent the mean ± SD. *P<0.05; **P<0.01. **e** Colony formation of CMT7364 and CIPp cells. Cells were treated with 8 μM ivermectin for 24 h or 48 h, followed by crystal violet staining of attached cells after 10 days. Triplicate wells were used for each treatment

To ascertain the antiproliferative effect of ivermectin in canine mammary cancer, the CCK-8 assay was used to assess the growth of canine mammary tumor cell lines (CMT7364 and CIPp) following ivermectin treatment. Ivermectin treatment decreased the cell viability of canine mammary tumor cell lines in a dose- and time-dependent manner (Fig. 1a and b). MDCK cell viability was also decreased following ivermectin treatment (Fig. 1c), but it was significantly higher than the canine mammary tumor cell lines following a 24-h exposure to 8 μM and 12 μM ivermectin (*P* < 0.01) (Fig. 1d). Next, the long-term effects of ivermectin on tumor cell proliferation were evaluated by colony formation assay. Similarly, ivermectin significantly reduced clonogenic survival in CMT7364 and CIPp cells (Fig. 1e). Taken together, these data show that ivermectin can exert an anti-proliferative effect on canine mammary tumor cells.

### Growth inhibition induced by ivermectin does not involve apoptosis

**Fig. 2:**
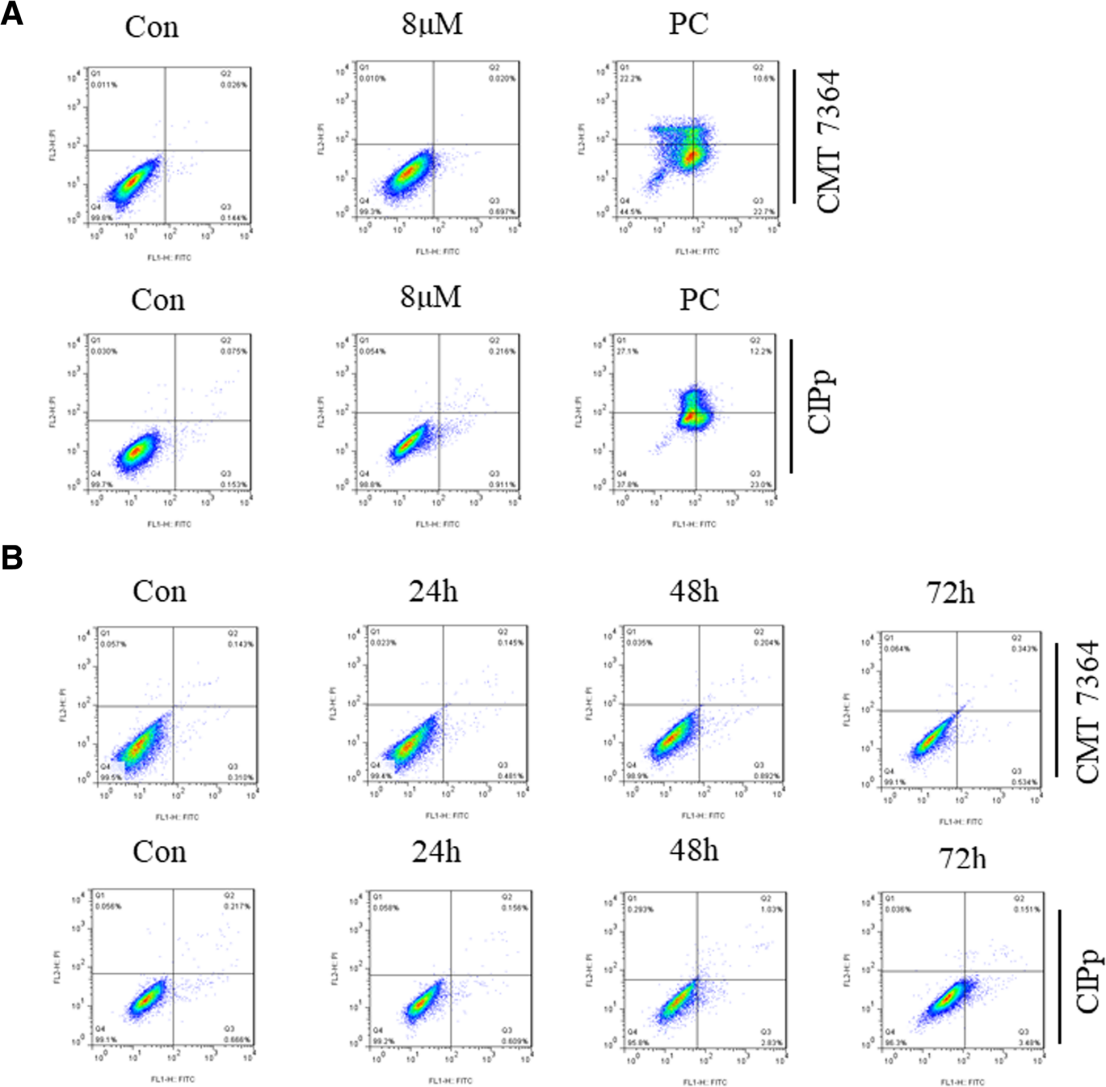
Flow cytometric dot plot analysis of Annexin V/PI staining in cells. **a** CMT7364 and CIPp cells were treated with 8 μM ivermectin for 48 h. PC, Apoptosis Inducer Kit (C0005, Beyotime, China), positive control. **b** CMT7364 and CIPp cells were treated with 12 μM ivermectin for 24 h, 48 h and 72 h. Triplicate wells were used for each treatment

Apoptosis is a major cause of viability inhibition induced by conventional anticancer therapies [15]. To determine whether ivermectin inhibited canine mammary tumor cell proliferation through the induction of apoptosis, we evaluated the apoptotic rate by Annexin V-PI staining. There was no significant effect on apoptosis in either canine mammary tumor cell line at 8 μM ivermectin treatment for 48 h (Fig. 2a). Even a 72-h exposure to 12 μM ivermectin did not dramatically alter apoptosis (Fig. 2b). These data indicate that ivermectin-induced growth inhibition is independent of apoptosis in these mammary cancer cell lines.

### Ivermectin induces cell cycle arrest at G1 phase

**Fig. 3:**
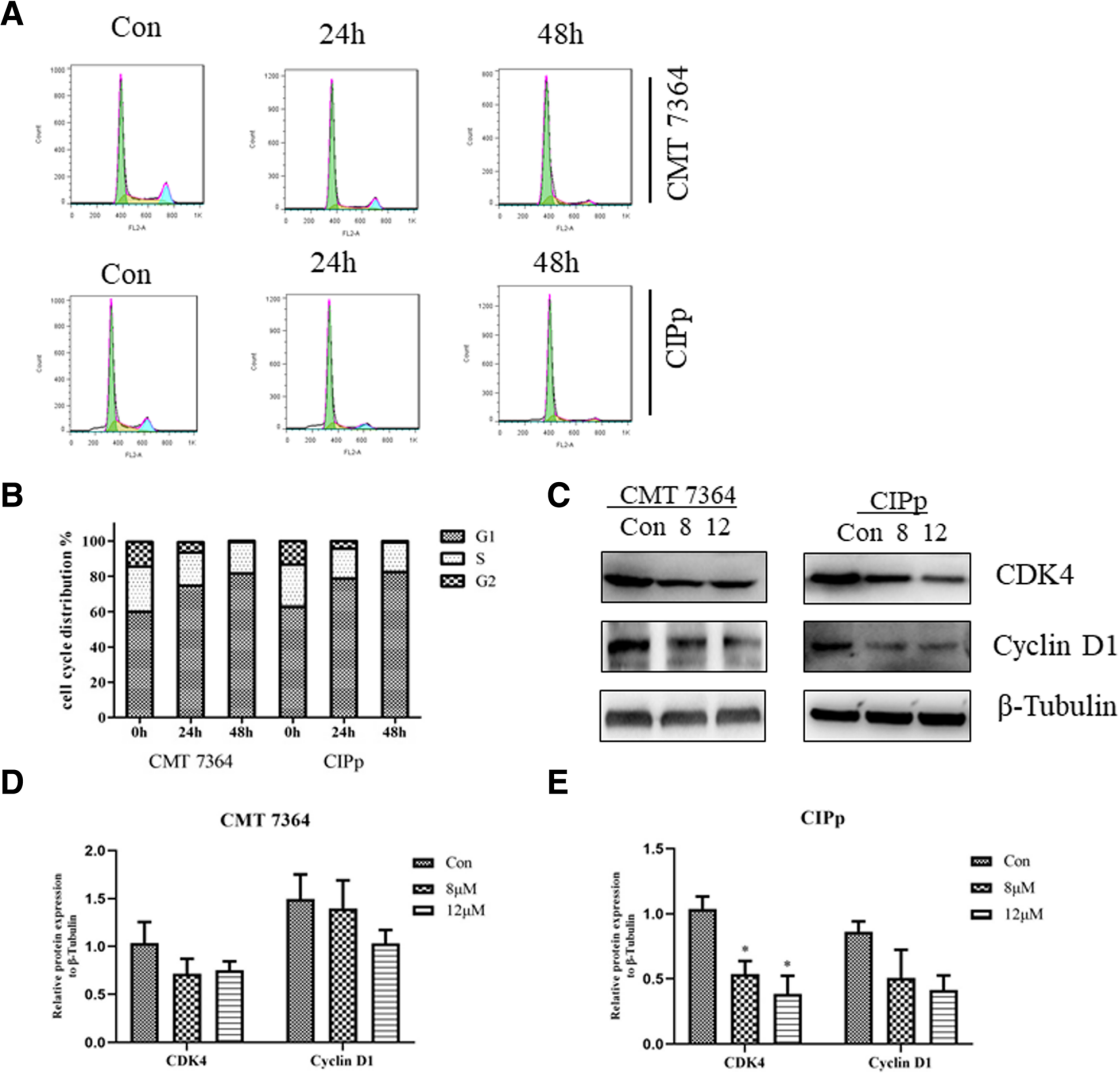
Effects of ivermectin on cell cycle distribution of canine mammary tumor cells. **a** Cell cycle distribution was analyzed by flow cytometry after 24 h or 48 h treatment with 8 μM ivermectin. **b** Cell cycle profiles of CMT7364 and CIPp cells after 24 h or 48 h treatment with 8 μM ivermectin. Data represent the mean. Triplicate wells were used for each treatment. **c** Western blotting showed the expression of CDK4 and cyclin D1 after 24 h treatment with ivermectin at 8 μM or 12 μM. Similar results were obtained from triplicate wells. Quantitative analysis of CMT 7364 (**d**) and CIPp (**e**) in **c**. Data represent the mean ± SD. *P<0.05; **P<0.01

To further explore the mechanisms involved in the effect of ivermectin on cell proliferation, we measured cell cycle distribution by flow cytometry. Ivermectin treatment resulted in partial G1 cell cycle arrest in both canine mammary tumor cell lines (Fig. 3a). Distribution of cells in G1 was increased after treatment with ivermectin in a time-dependent manner (Fig. 3b). Most of the mitogenic pathways result in the transcriptional induction of D-type cyclins and the subsequent activation of cyclin-dependent kinases (CDKs), such as cyclin D1 and CDK4, which can promote the G1-S cell cycle transition [16, 17]. We thus analyzed the expression of these proteins by western blot. As shown in Fig. 3c, expression of cyclin D1 and CDK4 were reduced in cell lines treated with ivermectin, and a significant difference was observed in the expression of CDK4 in the CIPp cell line (Fig. 3d and e). Based on these data, ivermectin induced cell cycle arrest at G1 phase through downregulating the expressions of cyclin D1 and CDK4.

### Ivermectin regulates the expression and nuclear translocation of β-catenin

**Fig. 4:**
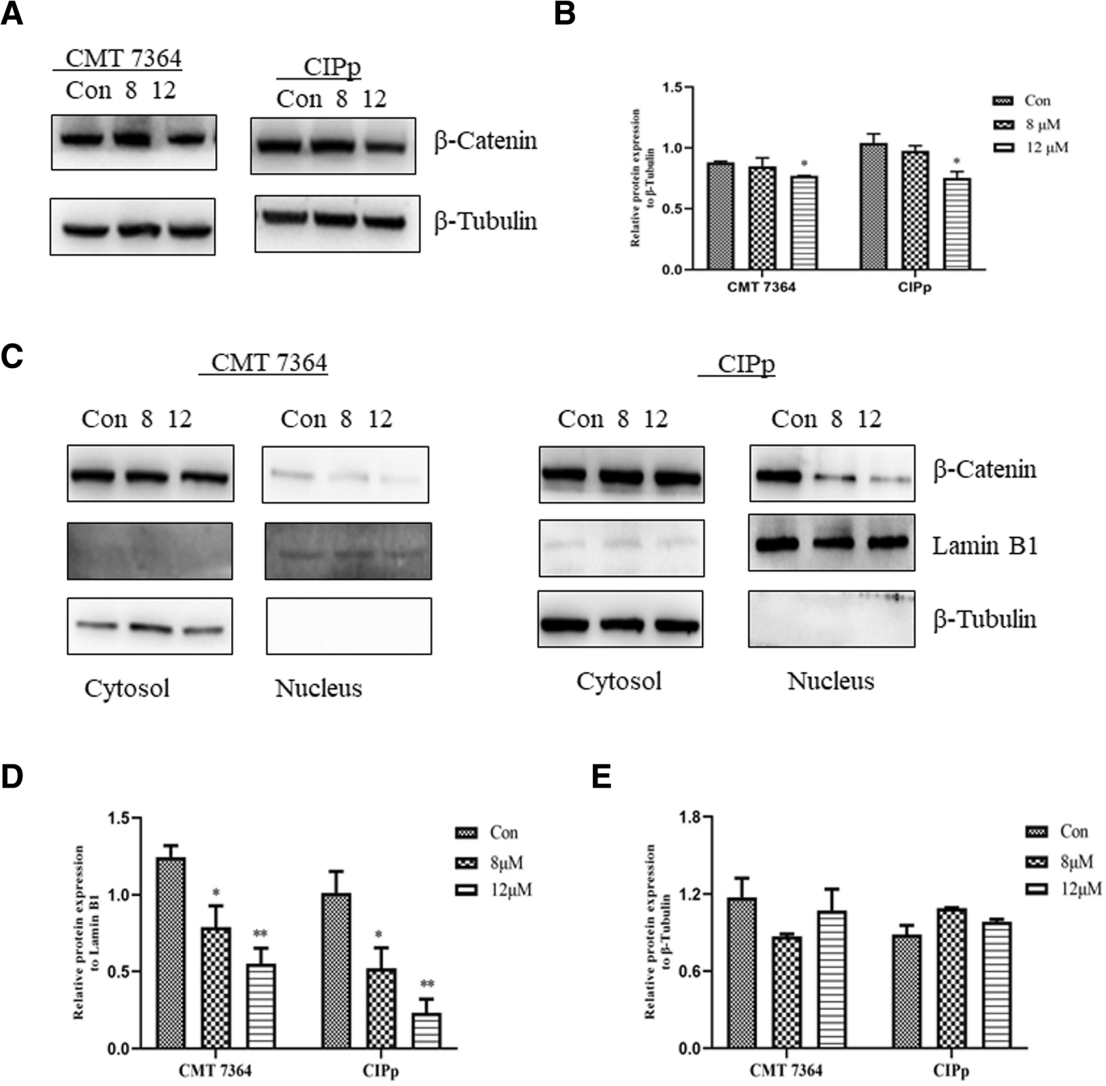
Effects of ivermectin on WNT signalling in canine mammary tumor cells. **a** Western blot showing expression of total β-catenin. Similar results were obtained from triplicate wells. **b** Quantitative analysis of CMT 7364 and CIPp in **a**. Data represent the mean ± SD. *P<0.05; **P<0.01. **c** Western blot showing expression of cytosolic and nuclear β-catenin after 24 h treatment with 8 μM or 12 μM ivermectin. Similar results were obtained from triplicate wells. Quantitative analysis of relative expression of β-catenin in nucleus (**d**) and cytosol (**e**) of CMT 7364 and CIPp in **c**. Data represent the mean ± SD. *P<0.05; **P<0.01

The activation of the WNT/β-catenin signaling pathway had been observed in many different cancers [18–20]. We measured expression of the key WNT pathway regulator, β-catenin, to determine whether ivemectin can impact this signalling pathway. As shown in Fig. 4a and b, the expression level of β-catenin was reduced significantly in each canine mamary tumor cell line at 12 μM ivermectin treatment for 24 h. In addtion, we found that nuclear expression of β-catenin was significantly reduced after treatment with ivermectin (Fig. 4d), but cytosolic expression did not change significantly (Fig. 4e). These data suggest that modulation of the WNT/β-catenin signaling pathway is associated with the effect of ivemectin on canine mammary tumor cells.

### Ivemectin suppresses CIPp xenograft tumor growth in vivo

**Fig. 5:**
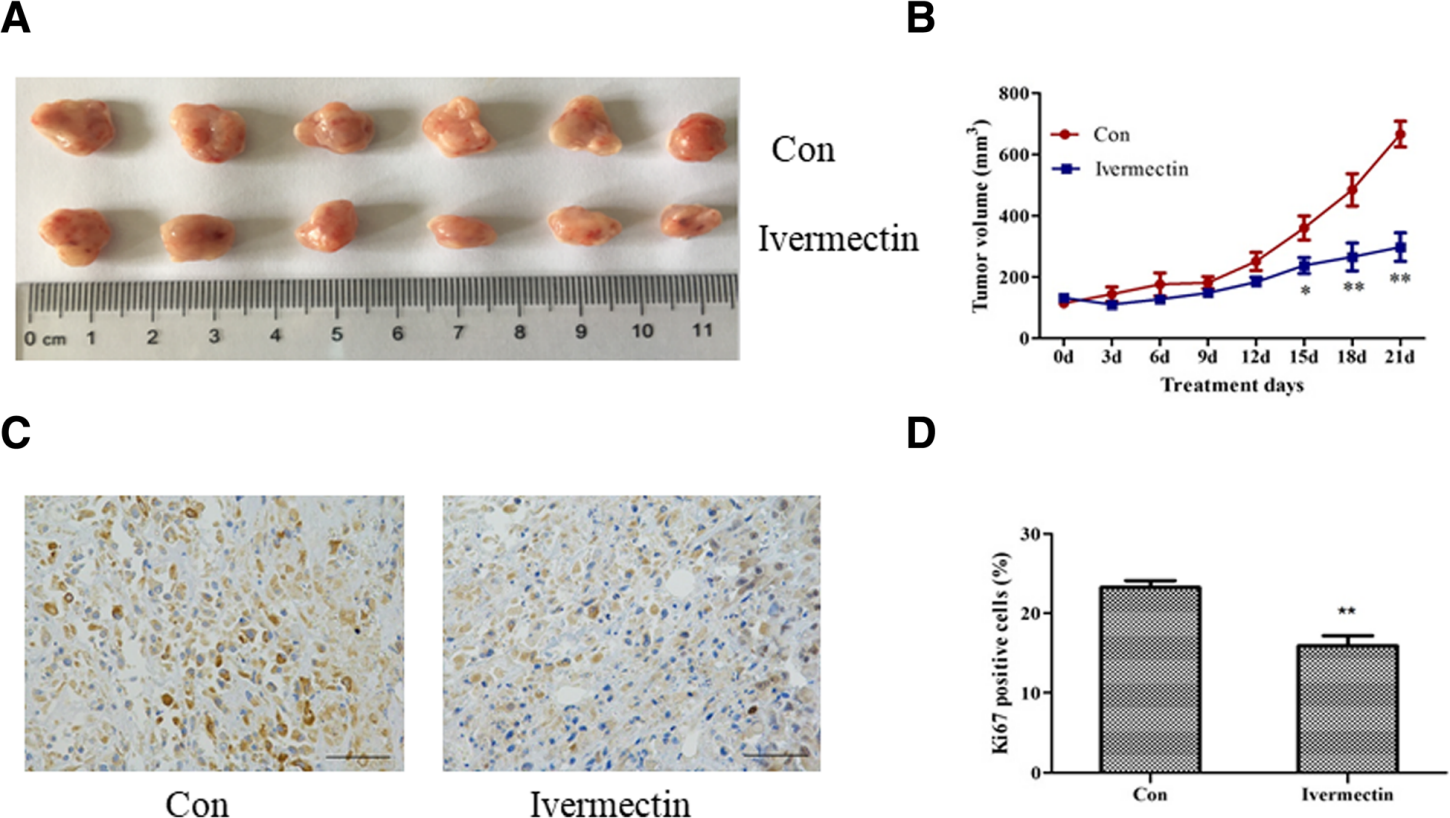
Ivermectin significantly inhibits CIPp tumor growth in vivo. **a** Representative photo of tumor at the end of the experiment. **b** Tumor volume. Data represent the mean ± SD with six mice per group. *P<0.05; **P<0.01. **c** Representative photos of IHC showing the expression of Ki67 (Scale bar =50 μm). **d** Quantitative analysis of Ki67 staining corresponding to the images in **c**. Data represent the mean ± SD with six mice per group. *P<0.05; **P<0.01

To evaluate the effect of ivermectin on canine mammary tumor growth in vivo, CIPp cells were injected subcutaneously into BALB/c nude mice to establish xenograft tumors. After 3 weeks of ivermectin administration by intraperitoneal injection, all xenograft tumors were collected (Fig. 5a and Additional file 1: Figure S1A). The volume of tumors in ivermectin treatment group was lower than that in the control group at the end of treatment (Fig. 5b and Additional file 1: Figure S1B). Furthermore, immunohistochemistry analysis with the proliferation marker Ki67 was performed in tumor tissues (Fig. 5c and Additional file 1: Figure S1C), and a significant difference was observed between these two groups (Fig. 5d and Additional file 1: Figure S1D) (*P*<0.01). These data were in concordance with our in vitro data, and confirmed the inhibition of tumor growth by ivermectin in canine mammary tumor cells.

### Ivermectin treatment does not cause systemic toxicity

**Fig. 6:**
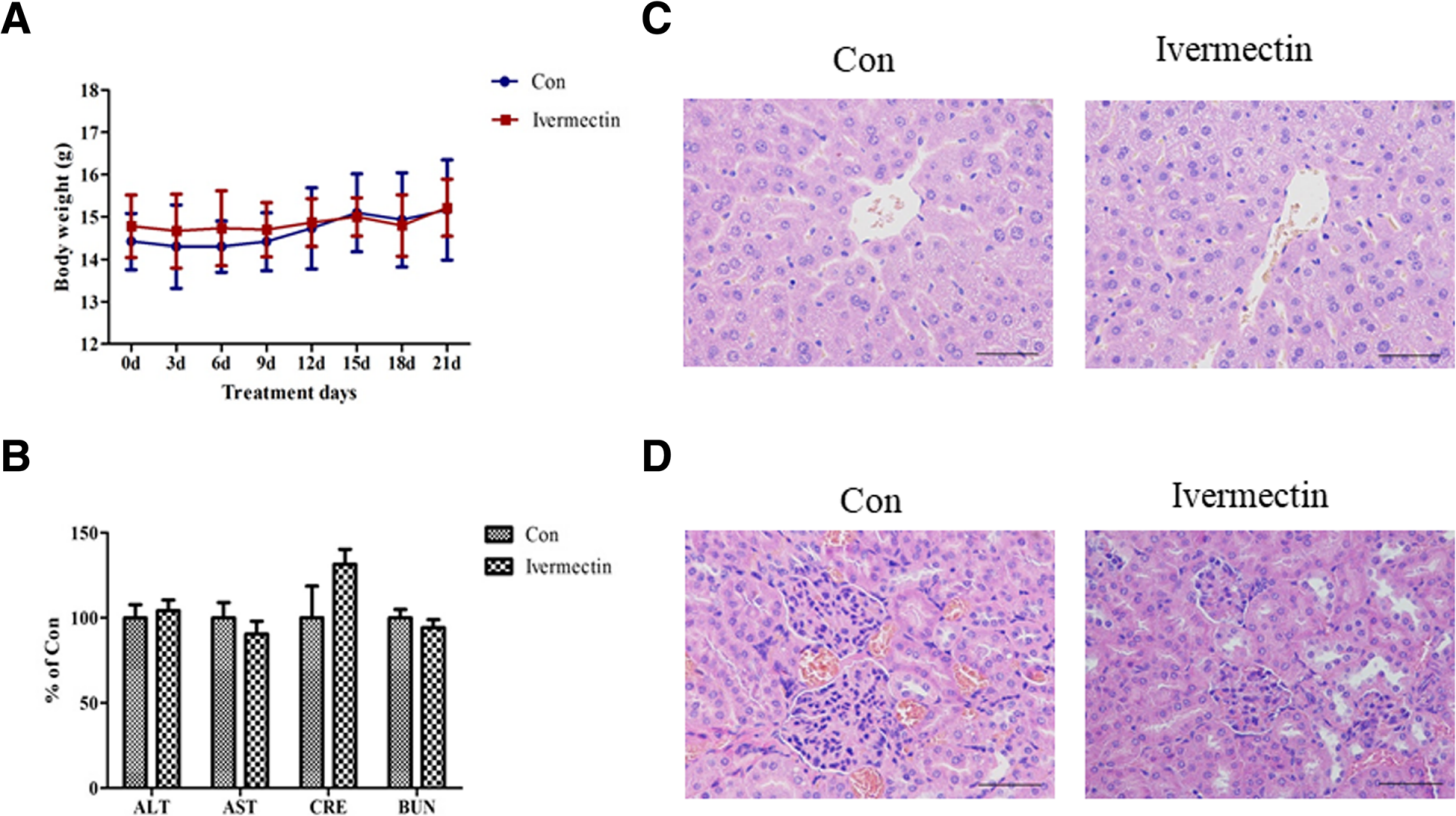
Ivermectin inhibits tumor growth with negligible toxicity. **a** mouse body weight throughout the duration of treatment. Data represent the mean ± SD with six mice per group. *P<0.05; **P<0.01. **b** serum AST, ALT, CRE and BUN from mice in different treatments. Data represent the mean ± SD with six mice per group. *P<0.05; **P<0.01. **c** Histological analysis of mouse liver tissue. Sections through the central veins of two classic lobules. Hepatocytes radiate as hepatic plates from the central vein. **d** Histological analysis of mouse kidney tissues. The renal tubules are lined by simple cuboidal epithelium. No lesions were found (Scale bar =50 μm)

To further confirm that ivermectin did not induce systemic toxicity compared with control-treated mice, body weights were measured every 3 days throughout the treatment. There was no significant weight loss in ivermectin-treatment group (Fig. 6a) (*P* > 0.05). At the end of treatment, we collected blood for major organ toxicity assessment. Levels of serum AST, ALT, CRE and BUN were measured for these two groups, and no significant differences were observed (Fig. 6b and Additional file 2: Figure S2B) (*P* > 0.05). Additionally, microscopic analysis of H&E-stained liver and kidney sections from these two groups showed no significant morphologic differences (Fig. 6c, d and Additional file 2: Figure S2C, D). These results indicated that ivermectin did not cause systemic toxicity.

## Discussion

Two canine mammary tumor cell lines were selected to demonstrate the efficacy of ivermectin in vitro. Ivermectin inhibited the proliferation of cancer cells (Fig. 1a and b) with less effects on a normal canine epithelial cell line (Fig. 1d), which indicates that ivermectin is preferentially inhibitory to the canine mammary tumor cell lines. Additionally, ivermectin dramatically inhibited colony formation in a time-dependent manner. In order to further determine the inhibitory effect of ivermectin on tumor growth in vivo, tumor inhibition experiments were performed in canine mammary tumor xenografts. Consistent with the in vitro results, ivermectin-treated xenografts showed significant growth suppression without significant changes in mouse body weight or hepatic/renal toxicity (Fig. 6). Consistent with previous studies [7–9], these data demonstrate that ivermectin is a potential anti-tumor agent in canine mammary tumor models.

This study also explored the mechanisms of action of ivermectin in canine mammary tumor cells. Inhibition of cell growth arises from a combination of increased apoptosis and/or inhibition of proliferation [21], so the effect of ivermectin in canine mammary tumor may due to induction of apoptosis. However, no significant apoptosis induction could be observed even with high concentrations of ivermectin for up to 72 h (Fig. 2), which was in agreement with previous work in human breast cancer [8]. In contrast, a study in glioblastoma cells showed that ivermectin significantly induced apoptosis in a dose-dependent manner [9]. These findings may be explained by the differences in different types of tumor. It is widely known that cell cycle checkpoints play a critical role in the progression of cancer, so cell cycle analysis was used to further explore the mechanisms of ivermectin-induced anti-proliferative activity. An increased percentage of cells in the G1 phase was observed (Fig. 3b), and this result was supported by the downregulated protein expression levels of key regulators of the G1 transition of the cell cycle, cyclin D1 and CDK4 (Fig. 3c, d and e). Several studies have confirmed that the cyclin D1-CDK4 complex is essential for G1 phase and to initiate proper transition to the S phase [22–24]. Taken together, these results demonstrate that ivermectin treatment of canine mammary tumor cells triggers accumulation of cells in the G1 phase via the inactivation of cyclin D1 and CDK4. Further studies should be performed to determine the exact mechanism of the reduction of cyclin D1 and CDK4 induced by ivermectin.

Since *CCND1* is one of the target genes of the WNT signaling pathway, and this signaling pathway has become a therapeutic target in various cancer types [25], we speculated that ivermectin treatment may have effects on WNT signaling. This assumption was confirmed by demonstrating reduced expression of β-catenin after ivermectin treatment (Fig. 4a and b). Furthermore, ivermectin significantly inhibited nuclear accumulation of β-catenin (Fig. 4d). β-catenin stabilization and nuclear translocation are essential to activate the WNT signaling [20]. These data indicate that the antitumor effect of ivermectin is likely to be mediated in part by inhibiting the nuclear translocation of β-catenin, downregulating WNT/β-catenin signaling. Further studies should be done to explore the exact mechanism of the translocation of β-catenin.

The doses of ivermectin used in mouse xenograft studies are variable [7–9, 26], from 5 to 40 mg/kg. In the present study, the dose of ivermectin was similar to that in Huang et al. [8], which utilized 0.12 mg ivermectin/mouse/day in a breast cancer model. This dose of ivermectin is higher than that commonly used in dogs, but appeared well tolerated in the mice based on lack of body weight loss, elevations in serum hepatic/renal function markers and absence of pathologic changes. Thus, higher doses than the antiparasitic dose used commonly in dogs may be tolerable. Caution should be made when select ivermectin as an anti-tumor drug for canine mammary tumor, since there is no references that show the dose necessary for the treatment of dogs is not toxic. Futher studies are needed to be find the exact concentration of ivermectin which has the anti-tumor effects in dogs without toxicity.

## Conclusion

This study is the first to evaluate the antitumor effect of ivermectin in canine mammary tumor cells in vitro and in xenograft models. Ivermectin’s antitumor effects are likely attributed to its ability to regulate cell cycle progression and WNT signaling. These findings support the investigation of ivermectin as a potential anti-cancer agent for canine tumor patients.

## Methods

### Cell lines and cell culture

The CMT7364 [27] (Veterinary Teaching Hospital, China Agricultural University, Beijing, China) and CIPp [28] (Graduate School of Agricultural and Life Sciences, University of Tokyo, Tokyo, Japan) canine mammary tumor cell lines, and Madin-Darby immortalized canine kidney (MDCK) cells (Cell bank of the Chinese Academy of Science, Beijing, China) were grown in DMEM (C11995500BT,Gibco, USA) medium with 10% fetal bovine serum (FBS) (16,000,044, Gibco, USA), and penicillin (100 units/mL) and streptomycin (0.1 mg/mL) (C0222, Beyotime, China). All cell lines were cultured in a humidified atmosphere with 5% CO_2_ at 37 °C.

### Cell proliferation evaluation

Cell viability was evaluated using a Cell Counting Kit-8 (CCK-8) (CK04, Dojindo Molecular Technologies, Inc., Kumamoto, Japan). CMT7364, CIPp and MDCK were plated in 96-well plates at 1 × 10^4^ cells per well and incubated overnight to allow attachment. Cells were treated with solvent (DMSO) (D2650, Sigma, USA) alone (control) or with different concentrations of ivermectin (I8898, Sigma, USA) (4, 8, 12, 20 and 40 μM). After 0, 12, 24 or 48 h, cell viability was assessed with CCK-8 according to the manufacturer’s instructions, measuring the optical density (OD) with a microplate reader (ELx808™; BioTek Instruments, Inc., Winooski, VT, USA) at 450 nm.

For the colony formation assay, CMT7364 and CIPp cells in single-cell suspension with ivermectin (8 μM) were plated in 6-well plates at 2000 cells per well. After 24 h and 48 h treatment, the plates were washed and fresh medium without ivermectin was added, followed by a10-day incubation. The attached cells were then stained with 0.1% (W/V) crystal violet (G1063, Solarbio, China) and the wells photographed.

### Apoptosis assay

Cells were plated in 6-well plates at 2 × 10^5^ cells per well, allowed to attach overnight, and treated with different concentrations of ivermectin. Cell apoptosis was detected by an annexin V-FITC/propidium iodide (PI) apoptosis detection kit (C0080, BD, USA) according to the manufacturer’s instructions, using a FACSCalibur flow cytometer (BD Biosciences) and FlowJo software (Version 7.6.1; Ashland, USA).

### Cell cycle analysis

Cells were treated as described in the apoptosis assay. After treatment, cells were washed twice with ice-cold PBS (SH30256, Hyclone, USA), fixed in 70% ethanol (AP0000008, i-presci, China) at 4 °C, treated with 50 μg/mL PI (C0080, Solarbio, China) for 15 min in the dark at room temperature. Then cells were detected with a BD FACSCalibur flow cytometer using selective gating to exclude doublets of cells. Data were analyzed using FlowJo software.

### Western blotting

Cells were plated in a 6-well plate at 2 × 10^5^ cells per well and treated with ivermectin (8 μM or 12 μM) or solvent alone (control). After treatment, protein extraction was performed with ice-cold lysis buffer (P0013B, Beyotime, China) except β-catenin in cytosol and nucleus, which was extracted with Minute Cytoplasmic and Nuclear Extraction Kits (SC-003, Invent Biotechnologies, Plymouth, MN, USA) following the manufacturer instructions, and then proteins were quantified using the BCA protein assay kit (P0012S, Beyotime, China). Equivalent samples (20 μg protein per lane) were subjected to SDS-PAGE on a 10% gel, then electrotransferred onto polyvinylidene fluoride (PVDF) membranes (IPVH000 10, Merck Millipore). The membranes were incubated with the following primary antibodies overnight at 4 °C: Cyclin D1 (MA5–12699, Thermo Fisher Scientific, USA, 1:1000), CDK4 (11026–1-AP, Proteintech, China, 1:1000), β-catenin (51067–2-AP, Proteintech, China, 1:1000), Lamin B1 (12987–1-AP, Proteintech, China, 1:1000) or β-Tubulin (T0198, Sigma, USA, 1:2000). As secondary antibodies, HRP-coupled anti-rabbit (SA00001–9, Proteintech, China, 1:2000) or anti-mouse (SA00001–1, Proteintech, China, 1:2000) were incubated for 1 h at room temperature, and finally exposed by a chemiluminescence imaging analysis system (Tanon 5200, China). Image J software (National Institutes of Health, Bethesda, MA, USA) was used to quantify the density of the bands.

### Mouse xenografts

Tumor xenografts were established in 5-week-old BALB/c nude mice (Vital River, China) by subcutaneous injection of CIPp cells into the mammary fat pad. For each tumor, 5 × 10^6^ cells were resuspended in 100 μL of PBS. The sixth day after inoculation, mice were treated with solvent alone (Control) (*n* = 6) or ivermectin (*n* = 6) via daily intraperitoneal injections. According to published literature [7–9, 17], the concentration of ivermectin used in xenograft models are variable in different tumors, ranging from 5 to 40 mg/kg. Given potential similarities between human breast tumor and canine mammary tumor [2], we injected ivermectin at 0.1 mg per mouse (equivalent to approximately 6–7 mg/kg) as performed in human breast tumor [8]. Tumor growth (tumor length and width) and body weights were measured every 3 days until the study was terminated on day 27. Tumor volume was calculated using formula: length x width^2^ / 2. At the end of the experiment, all the mice were first anesthetized with isofluorane and then euthanized via CO_2_ asphyxiation for collection of xenograft tumors, livers and kidneys. All animal procedures were approved by the Institutional Animal Care and Use Committee of China Agricultural University.

### Immunohistochemical analysis

CIPp xenograft tumors were dissected and fixed with10% (v/v) neutral-buffered formalin (G2161, Solarbio, China), embedded in paraffin wax and sectioned serially at 3 μm. After deparaffination and antigen retrieval with EDTA Antigen Retrieval Solution (G203, Epsilon, China), the tumor sections were incubated overnight at 4 °C with primary antibody for the proliferation marker protein antigen identified by monoclonal antibody Ki-67 (Ki67) (27309–1-AP; Proteintech, China, 1:1000). The biotinylated secondary antibody, anti-rabbit antibody IgG (ZB-2010, ZSGB-BIO, China), was incubated for 1 h at room temperature. Then the sections were stained with diaminobenzidine (ZLI-9018, ZSGB-BIO, China) and counterstained with hematoxylin (C0107, Beyotime, China). Images were captured with a digital microscope, and the amounts of Ki67 positive cells and total cells per image were automatically calculated by color using Image-pro-plus (IPP) software (Media Cybernetics, Washington, USA). The ratio between Ki67 positive cells and total cells was defined as the percentage Ki67 positive cells.

### Toxicity analysis

To determine whether the selected dose of ivermectin was toxic to animals, liver and kidney tissue was removed for formalin fixation and paraffin embedding for hematoxylin/eosin (H&E) staining and microscopic examination, and blood was collected for analysis to determine changes in serum aspartate aminotransferase (AST), serum alanine aminotransferase (ALT), serum creatinine (CRE) and urea nitrogen (BUN) by Chemical Analyzer (Maxmat PL II, MAXMAT SA, French).

### Statistical analysis

Numerical results were expressed as mean or mean ± standard deviation. Significant differences among groups were determined by analysis of variance or one-way ANOVA using GraphPad Prism 5.0 (GraphPad Software, California, USA) or SPSS18.0 (Statistical Product and Service Solutions, Chicago, USA). Differences were regarded significant at *P* < 0.05.

## Additional files

Additional file 1: **Figure S1.** Ivermectin significantly inhibits CIPp tumor growth in vivo. (A) Representative photo of tumor at the end of the experiment. (B) Tumor volume. Data represent the mean ± SD with six mice per group. *P<0.05; **P<0.01. (C) Representative photos of IHC showing the expression of Ki67 (Scale bar =50 μm). (D) Quantitative analysis of Ki67 staining corresponding to the images in C. Data represent the mean ± SD with six mice per group. *P<0.05; **P<0.01. (TIF 404 kb)

Additional file 2: **Figure S2.** Ivermectin inhibits tumor growth with negligible toxicity. (A) mouse body weight throughout the duration of treatment. Data represent the mean ± SD with six mice per group. *P<0.05; **P<0.01. (B) serum AST, ALT, CRE and BUN from mice in different treatments. Data represent the mean ± SD with six mice per group. *P<0.05; **P<0.01. (C) Histological analysis of mouse liver tissue. Sections through the central veins of two classic lobules. Hepatocytes radiate as hepatic plates from the central vein. (D) Histological analysis of mouse kidney tissues. The renal tubules are lined by simple cuboidal epithelium. No lesions were found (Scale bar =50 μm). (TIF 496 kb)

## Data Availability

The datasets used and analysed during the current study are available from the corresponding author on reasonable request.

## Abbreviations

ALT: Alanine aminotransaminase
AST: Aspartate aminotransaminase
BUN: Urea nitrogen
CCK-8: Cell Counting Kit-8
CDK4: Cyclin-dependent kinase 4
CRE: Creatinine
DAB: Diaminobenzidine
DMEM: Dulbecco’s modified Eagle’s medium
DMSO: Dimethyl sulphoxide
FBS: Fetal bovine serum
H&E-stained: Hematoxylin-eosin stained
HRP: Horseradish peroxidase
IPP: Image-pro-plus
Ki67: Nuclear-associated antigen
MDCK: Madin-Darby immortalized canine kidney
OD: Optical density
PBS: Phosphate buffered saline
PI: Propidium iodide
PVDF: Polyvinylidene fluoride
SDS-PAGE: Sodium dodecyl sulfate polyacrylamide gel electrophoresis
SPSS: Statistical Product and Service Solutions
WNT: Wingless-type MMTV integration site family
