# Supplementary figures and images for: Ivermectin inhibits canine mammary tumor growth by regulating cell cycle progression and WNT signaling
Source: BMC Vet Res. 2019 Aug 2;15:276. doi: 10.1186/s12917-019-2026-2 (PMC6679554; doi:10.1186/s12917-019-2026-2)

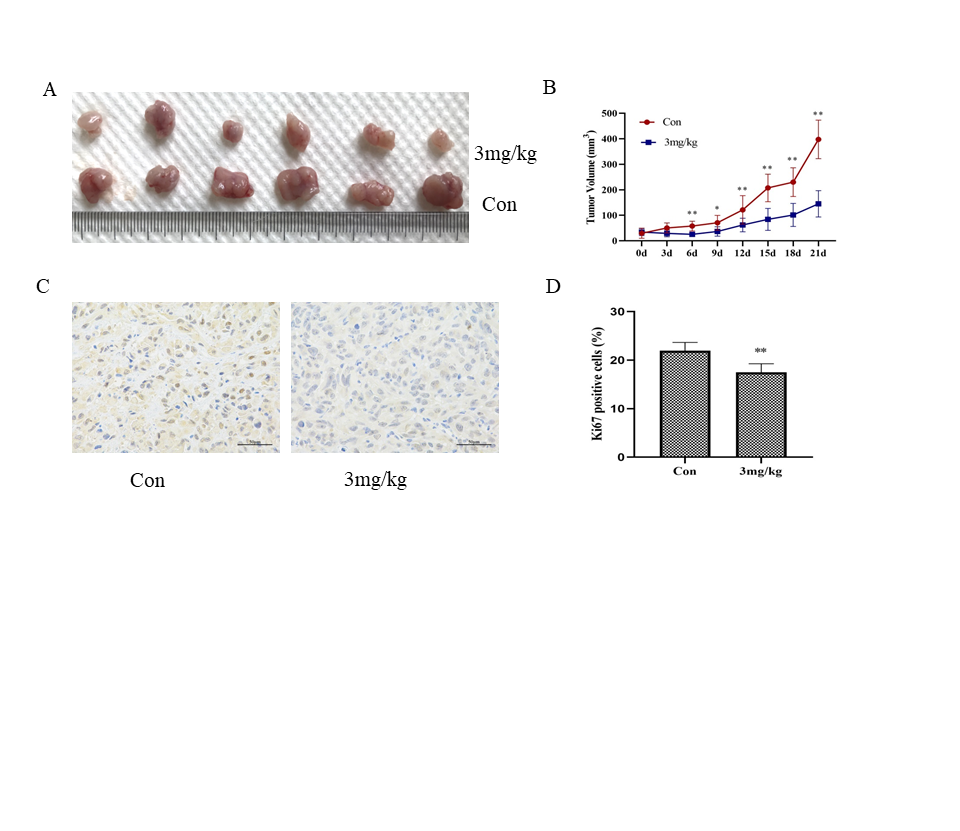

Supplement: Supplementary file 1 — Figure S1. Ivermectin significantly inhibits CIPp tumor growth in vivo. (A) Representative photo of tumor at the end of the experiment. (B) Tumor volume. Data represent the mean ± SD with six mice per group. *P<0.05; **P<0.01. (C) Representative photos of IHC showing the expression of Ki67 (Scale bar =50 μm). (D) Quantitative analysis of Ki67 staining corresponding to the images in C. Data represent the mean ± SD with six mice per group. *P<0.05; **P<0.01. (TIF 404 kb) [file 12917_2019_2026_MOESM1_ESM.tif]

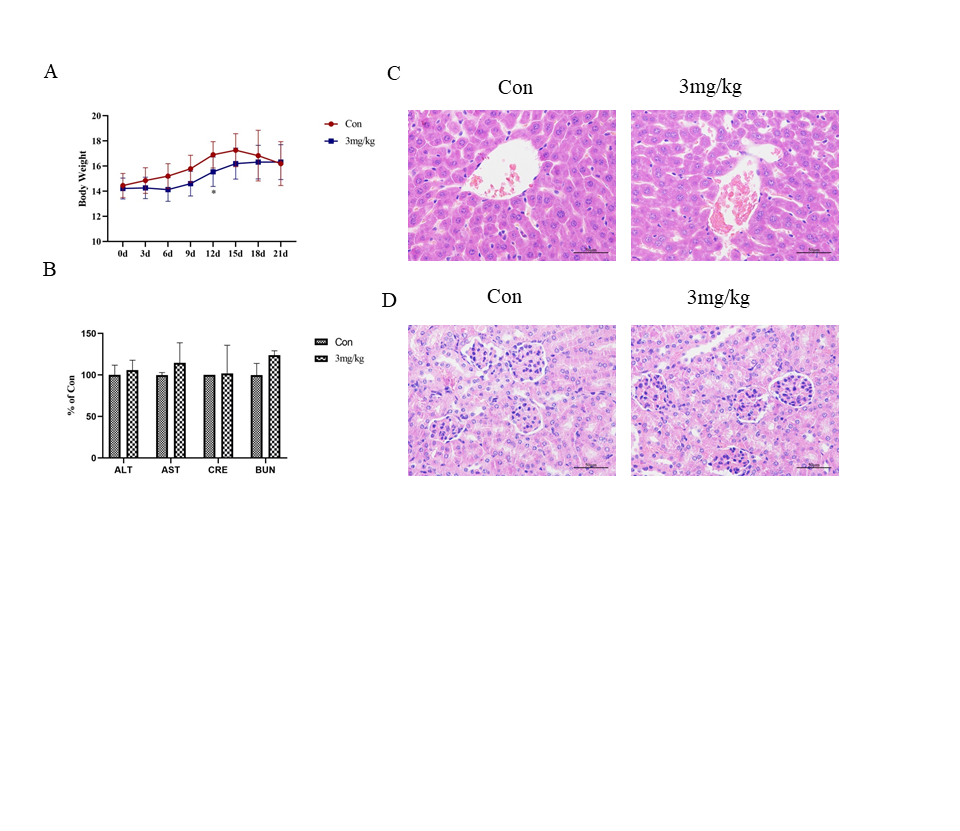

Supplement: Supplementary file 2 — Figure S2. Ivermectin inhibits tumor growth with negligible toxicity. (A) mouse body weight throughout the duration of treatment. Data represent the mean ± SD with six mice per group. *P<0.05; **P<0.01. (B) serum AST, ALT, CRE and BUN from mice in different treatments. Data represent the mean ± SD with six mice per group. *P<0.05; **P<0.01. (C) Histological analysis of mouse liver tissue. Sections through the central veins of two classic lobules. Hepatocytes radiate as hepatic plates from the central vein. (D) Histological analysis of mouse kidney tissues. The renal tubules are lined by simple cuboidal epithelium. No lesions were found (Scale bar =50 μm). (TIF 496 kb) [file 12917_2019_2026_MOESM2_ESM.tif]
